# Predictive Capability of an iPad-Based Medical Device (me*d*x) for the Diagnosis of Vertigo and Dizziness

**DOI:** 10.3389/fneur.2018.00029

**Published:** 2018-02-27

**Authors:** Katharina Feil, Regina Feuerecker, Nicolina Goldschagg, Ralf Strobl, Thomas Brandt, Albrecht von Müller, Eva Grill, Michael Strupp

**Affiliations:** ^1^Department of Neurology, University Hospital, Ludwig-Maximilians-Universität München, Munich, Germany; ^2^German Center for Vertigo and Balance Disorders, University Hospital, Ludwig-Maximilians-Universität München, Munich, Germany; ^3^Institute for Medical Information Processing, Biometrics and Epidemiology (IBE), University Hospital, Ludwig-Maximilians-Universität München, Munich, Germany; ^4^Institute for Clinical Neurosciences, University Hospital, Ludwig-Maximilians-Universität München, Munich, Germany; ^5^Parmenides Foundation, Pullach im Isartal, Germany

**Keywords:** vestibular, diagnosis, iPad, dizziness, vertigo, medical devices

## Abstract

**Background:**

Making the correct diagnosis of patients presenting with vertigo and dizziness in clinical practice is often challenging.

**Objective:**

In this study we examined the performance of the iPad based program me*d*x in the prediction of different clinical vertigo and dizziness diagnoses and as a diagnostic tool to distinguish between them.

**Patients and methods:**

The data collection was done in the outpatient clinic of the German Center of Vertigo and Balance Disorders. The “gold standard diagnosis” was defined as the clinical diagnosis of the specialist during the visit of the patient based on standardized history and clinical examination. Another independent and blinded physician finalized each patient’s case in the constellatory diagnostic system of me*d*x based on an algorithm using all available clinical information. These diagnoses were compared to the “gold standard” by retrospective review of the charts of the patients. The accuracy provided by me*d*x was defined as the number of correctly classified diagnoses. In addition, the probability of being test positive when a disease was present (sensitivity), of being test negative when a disease was absent (specificity), of having the disease when the test is positive (positive predictive value) and of not having the disease when the test is negative (negative predictive value) for the most common diagnoses were reported. Sixteen possible different vertigo and dizziness diagnoses could be provided by me*d*x.

**Results:**

A total of 610 patients (mean age 58.1 ± 16.3 years, 51.2% female) were included. The accuracy for the most common diagnoses was between 82.1 and 96.6% with a sensitivity of 40 to 80.5% and a specificity of more than 80%. When analyzing the quality of me*d*x in a multiclass problem for the six most common clinical diagnoses, the sensitivity, specificity, positive and negative predictive values were as follows: Bilateral vestibulopathy (81.6, 97.1, 71.1, and 97.5%), Menière’s disease (77.8, 97.6, 87.0, and 95.3%), benign paroxysmal positional vertigo (61.7, 98.3, 86.6, and 93.4%), downbeat nystagmus syndrome (69.6, 97.7, 71.1, and 97.5%), vestibular migraine (34.7, 97.8, 76.1, and 88.3%), and phobic postural vertigo (80.5, 82.5, 52.5, and 94.6%).

**Conclusion:**

This study demonstrates that me*d*x is a new and easy approach to screen for different diagnoses. With the high specificity and negative predictive value, the system helps to rule out differential diagnoses and can therefore also lead to a cost reduction in the health care system. However, the sensitivity was unexpectedly low, especially for vestibular migraine. All in all, this device can only be a complementary tool, in particular for non-experts in the field.

## Introduction

Vertigo and dizziness are among the most common symptoms in clinical practice and emergency rooms with a lifetime prevalence of between 20 and 30% (1, 2) and an annual incidence of about 11% (3). These symptoms are also the single most frequent complaint among patients older than 75 years (4, 5). A population-based study showed that within 1 year 10% of patients between 18 and 74 years consulted a doctor because of vertigo and dizziness (6).

The two most important keys leading to a correct diagnosis are a careful patient history and a systematic clinical neuro-otological, neuro-ophthalmological, and neurological examination (1, 7). Once the correct diagnosis is made in most cases—depending on the cause—specific effective treatment is available (1, 7, 8) either with drugs (9) or physio- and psychotherapy (1, 7). However, in clinical practice only a minority of all cases are correctly diagnosed (6): between 40 and 80% of patients do not get a specific diagnosis over a long period of time without receiving an adequate therapy (6, 10).

Therefore, the quality of life of affected patients is low with a high individual impairment (5, 11). The complaint is incapacitating and leads to decreased productivity and increased risk of clinical depression, falls, and injuries (12).

Patients with vertigo and dizziness also often consult doctors from different medical disciplines (e.g., neurology, ENT, internal medicine, orthopedics, or ophthalmology) (10). This process is accompanied by partly unnecessary laboratory examinations and imaging, such as a MRI of the cervical spine. For instance, >80% of the patients presenting at a tertiary dizziness center had at least one MRI imaging (10). This also causes high costs (13) with a financial burden for the health care system (5, 10, 11).

One reason for such incorrect and inadequate medical care in vertigo and dizziness patients could be the ambiguity of the terms themselves (1, 14). There are various meanings both on the side of the patients and on the side of different medical specialty doctors. Therefore, it is very import to clarify the patient’s actual complaint and to take the medical history carefully, systematically, and completely (14). To solve this problem, there have already been attempts at creating algorithms for diagnosing vertigo and dizziness more easily and correctly. One approach in the past has been the usage of standardized questionnaires. The predicted diagnoses from the questionnaire were compared with the ultimate clinical diagnosis made by an expert neuro-otologist based on clinical history, examination, and diagnostics (12). A subset of 47 questions was designed under multinomial logistic regression with high predictive accuracies for diagnosing vestibular migraine (92%), benign paroxysmal positional vertigo (90%), and Menière’s disease (86%) and with lower predictive accuracies for diagnosing vestibular neuritis (63%) (12). A smaller subset of 32 questions gave an overall predictive accuracy of 71% for these diagnoses (12).

Another approach of a database of vertigo patients involved a linear discrimination analysis using case history investigated in 1996 was able to obtain 90% prediction accuracy in classifying patients in the six most common diseases involving vertigo, namely benign paroxysmal positional vertigo, Menière’s disease, vestibular schwannoma, vestibular neuritis, sudden deafness, and traumatic vertigo (15). Based on these data (15), this group developed an otoneurological expert system (ONE) to aid the diagnostics of vertigo with a database containing patient’s history, clinical signs, and clinical test results necessary for a correct diagnosis (16, 17). In the validation of this expert system, the results showed that ONE solved 65% of the examined cases correctly compared with 69% by the physicians (16, 18). All these standardized databases and networks were compared with expert physicians who had made the diagnoses in advance independently of the unsupervised paradigm of the databases and networks.

In our study, we used a new system using an iPad-based program called me*d*x to systematically compare the prediction of clinical diagnoses in the neurological field of vertigo and dizziness and determine the possibilities of using this system to distinguish between different diagnoses of vertigo and dizziness. The aim of this study was to analyze the sensitivity, specificity as well as the positive predictive value (PPV) and negative predictive value (NVP) of the system regarding the correct diagnosis of vertigo disease entities.

## Materials and Methods

### Study Design and Subjects

The data collection was done in the outpatient clinic of the German Center for Vertigo and Balance Disorders in 2012 and 2013 (19). We conducted a retrospective study on a convenience sample of all patients consecutively referred to the center. We included patients aged over 18 years who had given informed consent. All patients received a complete standardized neurological and neuro-otological history, a systematic clinical neurological, neuro-ophthalmological, and neuro-otological examination. Furthermore, all patients were examined with bithermal caloric testing with video-oculography using the EyeSeeCam^®^. Depending on the patients’ complaints, further instrumental diagnostic testing was performed when necessary (particularly vestibular-evoked potentials, pure-tone audiometry, posturography, gait analysis). The patients were treated by a multidisciplinary team of experienced neurologists, ENT doctors, ophthalmologists, psychiatrists, and psychosomatic doctors. The authors were not involved in the clinical treatment of the patients.

### IPad-Based System (me*d*x)

**Figure 1:**
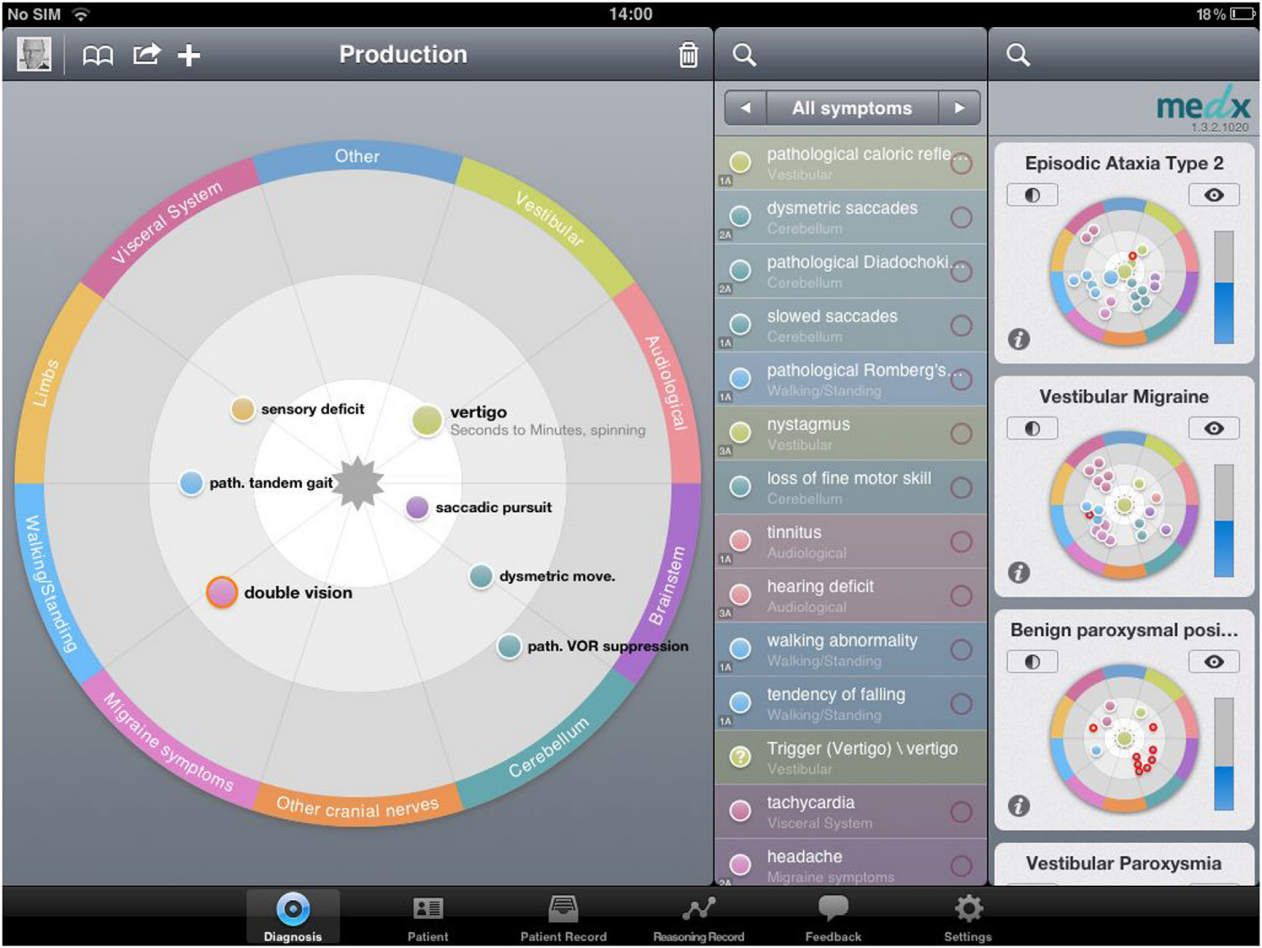
me*d*x iPad surface. Production of a diagnosis in constellatory diagnostic system, putting information of patient’s history. During the diagnostic process, the user has various choices, starting with patient history and symptoms, but other sources of information (clinical examination, diagnostic procedures) can be included as well (“all symptoms”). As this input is provided, the system, in parallel, already starts to seek and prioritize the best-matching patterns of symptoms (right part of the picture, diagnoses listed by probability). This process does not compare lists or maps of features, but it allows for the mutual unfolding of the meaning of all involved symptoms. The user can sort and differentiate the entered information according to the subjective (or objective) relevance; the closer the user “swipes” it to the center of the diagnostic field (on the left side of the picture), the higher this information is included in the calculation of the system. The more information you can add to the program, the higher the accuracy of the proposed diagnoses of the system.

The system is based on the recently developed methodology of “constellatory reasoning.” This is a computer decision-making algorithm. The mobile component is implemented on iPads. The repository of constellatory patterns resides on a central server on which the matching process and the collection and evaluation of data are also executed. The graphical user interface is optimized for intuitive access (see Figure 1). In “constellatory reasoning,” the physician represents the patient presentation directly as a constellation of symptoms done by finger swipes while talking to the patient. Other sources of information (clinical examination, diagnostic procedures) can be included as well. As this input is provided, the system, in parallel, already starts to seek and prioritize the best-matching patterns of symptoms. This process does not compare lists or maps of features, but it allows the mutual unfolding of the meaning of all involved symptoms. In doing so, it mimics the way experienced physicians actually think, and is therefore able to support the physician’s diagnostic reasoning in an effortless and “congenial” way. Due to this constellatory matching process, the system is also able to produce a continuously adapting list of useful next questions, clinical tests, or diagnostic examinations, prioritized according to the principle of “maximum information gain.” After finalizing the entry of clinical data, the most likely, probabilistically weighted diagnosis is shown to the physicians to support them in a diagnostic reasoning. Technically the system is based on a medical “description language” which has been developed specifically to bridge the gap between human thinking and machine-based operations. The medical “description language” characterization of a disease can be read, and thus controlled by human experts. At the same time, it is “readable” for the machine. The “reasoning operations” are based on a Boolean network that works in combination with a high-dimensional vector representation of both patients and diseases. The combination of the two methods aims to mimick what distinguishes good and experienced medical doctors from unexperienced doctors: the ability to appreciate the patient not as a list but as a constellation of symptoms, in which the individual symptoms are still capable of unfolding their meaning mutually.

### Outcome: Final Diagnosis––Gold Standard

The “gold standard” for the diagnosis of the patients presenting in the German Center for Vertigo and Balance Disorders was the clinical diagnosis of the attending physician during the visit of the patient in the outpatient clinic. This final diagnosis was made according to the proposed Classification of Vestibular Disorders of the Bárány Society and acted as the “gold standard.” Comparison with this defined “gold standard” allowed the diagnostic quality of the me*d*x system to be assessed. Therefore, the diagnosis of vestibular migraine was based on the diagnostic criteria of the Bárány Society (20), the diagnosis of Menière’s disease on the guidelines developed by the American Academy of Otolaryngology—Head and Neck Surgery, revised in 1995 (21), the diagnosis of bilateral vestibulopathy was defined by the criteria by Brandt et al. (22) and Kim et al. (23), the diagnosis of phobic postural vertigo was made according to the criteria defined by Dieterich et al. (24), and of benign paroxysmal positional vertigo based on Furman et al. (25). The diagnosis of cerebellar ataxia with neuropathy and bilateral vestibular areflexia syndrome (CANVAS) was based on the description of Szmulewicz et al. (26, 27). The other diagnoses were made by expert opinion and clinical reasoning. Vestibular neuritis was defined by the typical patient history and the clinical examination as well as a reduced caloric response (side difference > 25%) on the affected side and/or a pathological video-HIT (gain < 0.7). Typical symptoms are an acute onset of rotational/spinning vertigo, postural imbalance and nausea as well as a horizontal rotatory nystagmus beating toward the non-affected side. Furthermore, clinically there was no evidence for any central vestibular or ocular motor dysfunction (1). Downbeat nystagmus syndrome consisted of a spontaneous upward drift of constant velocity, increasing velocity or decreasing velocity, and a fast phase downward during gaze straight ahead with an increase of intensity during lateral and downward gaze. Additional ocular motor signs such as gaze-evoked nystagmus, deficient smooth pursuit eye movements, as well as deficient visual cancellation of the vestibular ocular reflex are often associated with DBN (1). Perilymph fistula and superior canal dehiscence syndrome are characterized by pressure-induced symptoms with attacks of vertigo, dizziness, and/or oscillopsia. In clinical examination, pressure-changing tests are positive. Radiological CT findings are important for diagnosing superior canal dehiscence syndrome; further diagnostic testing can be pathological c and/or oVEMP.

### Outcome: me*d*x Diagnosis

After the presentation of the patient, all the information taken by the responsible physician was entered in me*d*x directly on the iPad by an independent physician who was blinded to the patient’s diagnosis and did not know the patient. Other available sources of information (e.g., all clinical signs, diagnostic findings) were included as well. The independent and blinded physician finalized each patient’s case in the constellatory diagnostic system of me*d*x. Therefore, the physicians’ diagnostic decisions process and the algorithm input into the system were completely independent events. The me*d*x system finally assigned probabilities to each of the possible diagnoses. The most likely diagnosis for the individual patient is the first suggested diagnosis, the second likely the second suggestion, and so on.

There were 16 different diagnoses possible in the me*d*x constellatory diagnostic system. In the case of peripheral vestibular disorders, the possible diagnoses were as follows: Menière’s disease, benign paroxysmal positional vertigo, bilateral vestibulopathy, vestibular neuritis, vestibular paroxysmia, vestibular schwannoma, and perilymph fistula. In the case of central vestibular disorders, the possible diagnoses were as follows: vestibular migraine, downbeat nystagmus syndrome, cerebellar ataxia, CANVAS, episodic ataxia type 2, multiple system atrophy, progressive supranuclear palsy, and brainstem stroke. Furthermore, the diagnosis of phobic postural vertigo was possible. The diagnoses, such as CANVAS, cerebellar ataxia, and episodic ataxia type 2, had been pooled under the variable “Other Cerebellar Disorders” and the diagnoses vestibular paroxysmia, multiple system atrophy, vestibular schwannoma, perilymph fistula, progressive supranuclear palsy, and brainstem stroke under the variable “all others diagnoses” (others) due to a small number of cases. Thus, the diagnoses covered seven specific diagnoses and the two non-specific categories.

The diagnoses of the me*d*x system and the physicians were compared by a retrospective review of the charts of the patients over the study period.

### Ethical Standard

This study was carried out in accordance with the recommendations of the Institutional Review Board of the ethics committee of the Ludwig-Maximilian University Munich with written informed consent from all subjects. All subjects gave written informed consent in accordance with the 1964 Declaration of Helsinki and its later amendments. The protocol was approved by the Institutional Review Board of the ethics committee of the Ludwig Maximilian University Munich.

### Statistical Analysis

Categorical variables were described by absolute and relative frequencies and numeric variables by means and SD.

In order to evaluate the quality of the iPad-based system, we compared the first diagnoses suggested by me*d*x to the “gold standard” diagnoses of the experts at the German Center for Vertigo and Balance Disorders.

The main outcome was a multinomial variable with nine different categories (multiclass problem). Thus, the overall accuracy was defined as exact agreement of the first suggested me*d*x diagnosis and the final diagnosis. A patient was only classified correctly, if the me*d*x-diagnosis exactly matched the final diagnosis.

In order to report standard diagnostic parameters, i.e., accuracy, sensitivity, specificity, the PPV, and the NPV, each diagnosis was compared with all remaining diagnoses. This reduced the classification problem to a two-class problem (i.e., a “one versus all” approach). To put this into context, a patient was classified correctly if the me*d*x diagnosis exactly matched the final diagnosis or if the me*d*x diagnosis correctly excluded a certain diagnosis. Regarding the capability of the me*d*x system to diagnose Menière’s disease as an example, a patient with the me*d*x diagnosis of phobic postural vertigo would be treated as a Non-Menière case. If the final diagnosis was vestibular migraine, this would still be considered as a correct classification, because both the me*d*x system and the expert classified the patient as a non-Menière case (see Table 1). In general following the notation in Table 1, sensitivity is the probability of the classifier (the me*d*x system) classifying a patient as having a specific disease when this disease is truly present $\frac{A}{A+C}$; specificity is the probability of classifying a patient as not having a specific disease specific disease when this disease is truly absent $\frac{D}{B+D}$; PPV is the probability of having a specific disease when the me*d*x system classified a patient as having this disease $\frac{A}{A+B}$; NPV is the probability of not having a specific disease when the me*d*x system classified a patient as not having this disease $\frac{D}{C+D}$. As an overall measure we report the accuracy which is defined as the probability of being correctly classified $\frac{A+D}{A+B+C+D}$.

**Table 1:** Example for the calculation of diagnostic parameters for me*d*x system.

		Truth (expert opinion)	
		Meniere’s disease	Other diagnosis
Test result (me*d*x system)	Meniere’s disease	A	B
	Other diagnosis	C	D

In this example, accuracy was the overall number of correctly classified patients, while sensitivity is the percentage of patients classified as having Menière’s disease by the me*d*x system (A) among patients who truly had Menière’s disease (A + C). Specificity was the percentage of patients classified as a non-Menière patient by the me*d*x system (D) among patients who truly did not have Menière’s disease (B + D). PPV was the percentage of patients who truly had Menière’s disease (A) among patients who were classified as having Menière’s disease by the iPad system (A + B). NPV was the percentage of patients who truly did not have Menière’s disease (D), among patients who were classified as a non-MD patient by the iPad system (C + D). Diagnostic parameters were estimated using the R package “caret” (28) which was developed for the statistical package R.3.0.3 (29).

## Results

### Patients and Diagnoses Made by Physicians

In total, 610 patients presenting in the German Center for Vertigo and Balance Disorders (mean age: 58.1 ± SD 16.3; 51.2% female) were included. The most common clinical diagnoses were as follows: phobic postural vertigo (19.3%, mean age 49.8 ± 16.5, 57.5% female), Menière’s disease (17.7%, mean age 60.4 ± 14.4, 45.6% female), vestibular migraine (16.6%, mean age 48.7 ± 14.6, 78.4% female), and benign paroxysmal positional vertigo (15.4%, mean age 59.2 ± 13.5, 52.3% female) followed by bilateral vestibulopathy (8.0%, mean age 71.4 ± 13.3, 41.7% female) and downbeat nystagmus syndrome (7.54%, mean age 72.0 ± 9.8, 39.1% female). All other diagnoses accounted for 15.4% of cases (mean age 60.8 ± 14.8, 30.2% female) (for details of the represented diagnosis, see Table 2).

**Table 2:** Clinical characteristics of diagnosis of study collective.

	All	Phobic postural vertigo	MD	VM	BPPV	BVP	DBN	VN	Other cerebellar disorders	Others
*N*	610 100%	118 19.3%	108 17.7%	101 16.6%	94 15.4%	49 8.0%	46 7.5%	26 4.3%	26 4.3%	42 6.9%
Age	58.47 (SD: 15.82)	50.7 (SD: 15.51)	60.85 (SD: 13.33)	48.7 (SD: 14.64)	59.19 (SD: 13.45)	71.43 (SD: 13.25)	71.97 (SD: 9.78)	59.36 (SD: 14.22)	65.85 (SD: 13.79)	59.92 (SD: 14.67)
Female	293 (51.2%)	61 (57.5%)	47 (45.6%)	76 (78.4%)	45 (52.3%)	20 (41.7%)	18 (39.1%)	7 (26.9%)	6 (24%)	13 (37.1%)

*BPPV, benign paroxysmal positional vertigo; BVP, bilateral vestibulopathy; DBN, downbeat nystagmus syndrome; MD, Menière’s disease; VM, vestibular migraine; VN, vestibular neuritis. Other cerebellar disorders: Cerebellar ataxia with neuropathy and bilateral vestibular areflexia syndrome; cerebellar ataxia; episodic ataxia type 2. Others: all other diagnoses (vestibular paroxysmia, multiple system atrophy; vestibular schwannoma, perilymph fistula, progressive supranuclear palsy, brainstem stroke)*.

We performed the overall comparison of final diagnoses with diagnoses suggested by the system (multiclass problem) as well. The overall accuracy for the different diagnoses as a multiclass problem ranged from 82.1% (for phobic postural vertigo) to 96.6% (in vestibular neuritis and “other cerebellar disorders”) (for details, see Table 3).

**Table 3:** Comparison of the classification of me*d*x system and the truth as a multiclass problem for the six most prevalent diagnoses.

		Truth (expert opinion)													
		Phobic postural vertigo	MD	VM	BPPV	BVP	DBN	VN	Other cerebellar disorders	Others	Sensitivity (%)	Specificity (%)	PPV (%)	NPV (%)	Accuracy (%)
Test result (me*d*x system)	Phobic postural vertigo	95	1	2	1	1	2	1	0	15	80.5	82.5	52.5	94.6	82.1
	MD	9	84	4	3	3	0	2	0	3	77.8	97.6	87.5	95.3	94.1
	VM	49	8	35	2	0	0	1	0	6	34.7	97.8	76.1	88.3	87.4
	BPPV	5	0	0	58	1	1	2	1	26	61.7	98.3	86.6	93.4	92.6
	BVP	4	0	0	0	40	1	0	3	1	81.6	97.1	71.4	98.4	95.9
	DBN	7	1	1	0	1	32	0	3	1	69.6	97.7	71.1	97.5	95.6
	VN	2	2	1	2	2	1	11	0	5	42.3	99.0	64.7	97.5	96.6
	Other cerebellar disorders	3	0	2	0	3	5	0	12	1	46.2	98.8	63.2	97.6	96.6
	Others	7	0	1	1	5	3	0	0	25	59.5	89.8	30.1	96.8	87.7

*Benign paroxysmal positional vertigo, benign paroxysmal positional vertigo; BVP, bilateral vestibulopathy; DBN, downbeat nystagmus syndrome; MD, Menière’s disease; VM, vestibular migraine; VN, vestibular neuritis. Other cerebellar disorders: cerebellar ataxia with neuropathy and bilateral vestibular areflexia syndrome; cerebellar ataxia; episodic ataxia type 2. Others: all other diagnoses (vestibular paroxysmia; multiple system atrophy; vestibular schwannoma, perilymph fistula; progressive supranuclear palsy; brainstem stroke). Sensitivity: true positive rate, specificity: true negative rate. PPV, positive predictive value; NPV, negative predictive value*.

### Comparison of Final Diagnoses with Diagnoses Suggested by the System––Two-Class Problem

In the six most common clinical diagnoses in our patient collective the sensitivity, specificity, PPV, and NPV are described in detail in Table 3. The accuracy for the most common diagnoses was between 82.1 and 96.6% with a sensitivity of 40 to 80.5% and a specificity of at least 80%. Analyzing a less-frequent diagnosis such as vestibular neuritis sensitivity was 46.2% with a high specificity of 99.9%, a PPV of 64.7%, and NPV of 97.6%.

The accuracy for the eight most relevant diagnoses was high with 82.1% for phobic postural vertigo and 96.6% for cerebellar disorders (e.g., episodic ataxia type 2). The sensitivity differed from 34.7% in vestibular migraine and was the highest diagnosing phobic postural vertigo with a sensitivity of 80.5%.

Considering the results of the system as a two-class problem as described above, the accuracy of the first suggested diagnosis of the system with the final diagnosis of the physician ranged from 82 to 99% (see Table 4). The lowest accuracy was shown diagnosing phobic postural dizziness (82.1%) and vestibular migraine (87.4%). The sensitivity and specificity of each diagnosis differed. Taking into consideration only the first suggested diagnosis by the me*d*x system, diagnosing vestibular neuritis or vestibular migraine the sensitivity was considerably lower (42.3% for vestibular neuritis and 34.7% for vestibular migraine) than diagnosing phobic postural vertigo (80.5%), Menière’s disease (77.8%) or bilateral vestibulopathy (81.6%). On the other hand, the specificity of the correct diagnosis was still quite good ranging from 92 to 99% in all diagnoses (for further details, see Table 4).

**Table 4:** Statistical value analyzing me*d*x system in a one-class problem and taking into consideration the first diagnosis suggested by the me*d*x system.

Diagnosis made in outpatient clinic	Statistical value of first diagnosis suggested by me***d***x system				
	Sensitivity (%)	Specificity (%)	PPV (%)	NPV (%)	Accuracy (%)
Phobic postural vertigo	80.5	82.5	52.5	94.6	82.1
Menière’s disease	77.8	97.6	87.5	95.3	94.1
Vestibular migraine	34.7	97.8	76.1	88.3	87.4
Benign paroxysmal positioning vertigo	61.7	98.3	86.6	93.4	92.6
Bilateral vestibulopathy	81.6	97.1	71.4	98.4	95.9
Downbeat nystagmus	69.6	97.7	71.1	97.5	95.6
Vestibular neuritis	42.3	99.0	64.7	97.5	96.6
Other cerebellar disorders	46.2	98.8	63.2	97.6	96.6
Others	59.5	89.8	30.1	96.8	87.7

*PPV, positive predictive value; NPV, negative predictive value*.

Considering the first two diagnoses suggested by the system based on the medical decision language and taking these into consideration, the results of the predictive power of the system changed in the following way: first, the overall accuracy of the diagnosis was slightly lower with 95.0% compared with 95.5% in the first analysis. Comparing the diagnosis, there are no big changes except the accuracy of diagnosing phobic postural vertigo (accuracy changed from 82.1 to 74.3%) and vestibular paroxysmia (91 to 68.9%). Another noticeable and important effect is the changed sensitivity for diagnosing vestibular migraine from 34.7 to 64.4% (each with a specificity of >90%) just like in vestibular neuritis (sensitivity in the first analysis 42.3 to 69.2% in the second analysis) and cerebellar disease (sensitivity in the first analysis 21.1 to 47.4% in the second analysis). A high sensitivity and a high specificity were shown for Menière’s disease (sensitivity 85.2%, specificity 94%), bilateral vestibulopathy (sensitivity 87.8%, specificity 94.3%), and downbeat nystagmus syndrome (sensitivity 84.8%, specificity 94%). Compared with that, in particular the diagnosis of vestibular paroxysmia showed a much lower sensitivity (76.0%) as well as specificity (78.5%). For further details, see Table 5.

**Table 5:** Statistical value analyzing me*d*x system in a one-class problem and taking into consideration the first two diagnoses suggested by the me*d*x system.

Diagnosis made in outpatient clinic	Statistical value of the first two diagnosis suggested by me***d***x system				
	Sensitivity (%)	Specificity (%)	Positive predictive value (%)	Negative predictive value (%)	Accuracy (%)
Phobic postural vertigo	90.7	70.3	42.3	96.9	74.3
Menière’s disease	85.2	94	75.4	96.7	92.5
Vestibular migraine	64.4	91.6	60.2	92.8	87
Benign paroxysmal positioning vertigo	84	90.1	60.8	96.9	89.2
Bilateral vestibulopathy	87.8	94.3	57.3	98.9	93.8
Downbeat nystagmus	84.8	94	53.4	98.7	93.3
Vestibular neuritis	69.2	97.6	56.3	98.6	96.4
Other cerebellar disorders	73.1	92.6	30.6	98.7	91.8
Others	88.1	45.4	10.7	98.1	48.4

## Discussion

The major findings of this study were as follows.

First, this system allows a systematic acquisition of patients’ data in terms of patient history, clinical, and laboratory examinations. The use of a tablet PC has also been shown to be practical, easy, and intuitive as in other studies (30).

Second, the resulting predictive model correctly matched a patient to his ultimate clinical diagnosis 95.5% of the time over all different diagnoses (12). The results were different comparing the single diagnosis, but still showed a prediction of >80% in every single diagnosis. Compared with that, results from a patient’s history questionnaire with 47 variables correctly matched diagnosis in 84% (12). Another classification of patients on the basis of oto-neurological data by using Kohonen networks and compared with other neural networks showed an equally good classification analyzing benign paroxysmal positional vertigo, Menière’s disease, and vestibular neuritis with true positive rates >70% (31). In conclusion, the accuracy of diagnostic rules in another database was >90% (range 91–98%) for six otological diseases involving vertigo, except for Menière’s disease which had an accuracy of 81% (32).

Third, the accuracy of the system was high: >90% for the most common diagnoses except for vestibular migraine and phobic postural vertigo. An important result of the analysis is the high specificity and the high NPV of the different diagnoses of vertigo and balance disorders. This is particularly important because by using the system many diseases can be ruled out. As a result, an early exclusion of possible differential diagnoses by the me*d*x system has in turn consequences on the further implementation of diagnostic procedures. Other possible consequences are cost savings for the health system on the one hand and saving patients from a possible odyssey of unnecessary diagnostic procedures on the other hand. However, one had to admit that the sensitivity for vestibular migraine, vestibular neuritis, and “other cerebellar disorders” was quite low. In these diagnoses, it was obviously difficult to make a correct classification by the system comparing these results to the final diagnoses of the specialists. In the case of vestibular migraine, this could be partly explained by the fact that vestibular migraine is a diagnosis of exclusion (20). Furthermore, at the time of data collection the diagnostic criteria for vestibular migraine (20) were not in use, so this could also explain the difficulties of the me*d*x system to make the correct diagnosis.

All in all, compared with other predictive models (12, 16–18, 32), the accuracy of our new approach was higher (mean 95.5%), especially the predictive value of me*d*x constellatory diagnosis was also higher than in other expert systems taking into account patient history data and clinical vestibular functions.

This study has some methodological limitations. First, our analyses are based on the patients presenting in our patient clinic in a tertiary academic care center. Our sample of patients is not representative for the patient collective seen in other outpatients units or clinics. Second, the doctors were experts. Therefore, the next step should be to review the results in primary care (family doctors) and secondary care (ENT doctors and neurologists).

Despite these limitations and a low sensitivity for certain diagnoses such as vestibular migraine, this system could be used a complementary tool, in particular for non-experts.

## Ethics Statement

This study was carried out in accordance with the recommendations of the Institutional Review Board of the ethics committee of the Ludwig-Maximilian University Munich with written informed consent from all subjects. All subjects gave written informed consent in accordance with the 1964 Declaration of Helsinki and its later amendments. The protocol was approved by the Institutional Review Board of the ethics committee of the Ludwig-Maximilian University Munich.

## Author Contributions

KF contributed to the drafting/revising of the manuscript for content, including medical writing, study concept/design, interpretation of data, and acquisition of data. RF, TB, and MS contributed to the revising of the manuscript for content, including medical writing, study concept. NG contributed to the acquisition of data and revising of the manuscript. AM contributed to the development of the manuscript and software, and revising of the manuscript for content. RS and EG contributed to the statistical analysis, analysis and interpretation of data, and revising of the manuscript for content.

## Conflict of Interest Statement

The authors declare that the research was conducted in the absence of any commercial or financial relationships that could be construed as a potential conflict of interest. KF, RF, RS, AM, EG, and TB report no disclosures. NG received speaker’s honoraria from Actelion. MS is joint chief editor of the *Journal of Neurology*, editor in chief of *Frontiers of Neuro-otology*, and section editor of *F1000*. He has received speaker’s honoraria from Abbott, Actelion, Auris Medical, Biogen, Eisai, GSK, Henning Pharma, Interacoustics, MSD, Otometrics, Pierre-Fabre, TEVA, and UCB. He acts as a consultant for Abbott, Actelion, AurisMedical, Heel, IntraBio, and Sensorion.
